# Beneficial insects are associated with botanically rich margins with trees on small farms

**DOI:** 10.1038/s41598-021-94536-3

**Published:** 2021-07-26

**Authors:** Sarah E. J. Arnold, Filemon Elisante, Prisila A. Mkenda, Yolice L. B. Tembo, Patrick A. Ndakidemi, Geoff M. Gurr, Iain A. Darbyshire, Steven R. Belmain, Philip C. Stevenson

**Affiliations:** 1 Natural Resources Institute, University of Greenwich, Chatham Maritime, Kent, ME4 4TB UK; 2 The Nelson Mandela African Institution of Science and Technology, Arusha, Tanzania; 3 Department of Biology, The University of Dodoma, P.O. Box 338, Dodoma, Tanzania; 4 Biosciences Department, Sokoine University of Agriculture, P.O Box 3000, Morogoro, Tanzania; 5 Lilongwe University of Agricultural and Natural Resources, Bunda, Lilongwe, Malawi; 6 School of Agricultural and Wine Sciences, Charles Sturt University, Orange, NSW 2800 Australia; 7 Royal Botanic Gardens, Kew, Richmond, Surrey, TW9 3AE UK

**Keywords:** Agroecology, Biodiversity, Community ecology, Ecosystem services

## Abstract

Beneficial insect communities on farms are influenced by site- and landscape-level factors, with pollinator and natural enemy populations often associated with semi-natural habitat remnants. They provide ecosystem services essential for all agroecosystems. For smallholders, natural pest regulation may be the only affordable and available option to manage pests. We evaluated the beneficial insect community on smallholder bean farms (*Phaseolus vulgaris* L.) and its relationship with the plant communities in field margins, including margin trees that are not associated with forest fragments. Using traps, botanical surveys and transect walks, we analysed the relationship between the floral diversity/composition of naturally regenerating field margins, and the beneficial insect abundance/diversity on smallholder farms, and the relationship with crop yield. More flower visits by potential pollinators and increased natural enemy abundance measures in fields with higher plant, and particularly tree, species richness, and these fields also saw improved crop yields. Many of the flower visitors to beans and potential natural enemy guilds also made use of non-crop plants, including pesticidal and medicinal plant species. Selective encouragement of plants delivering multiple benefits to farms can contribute to an ecological intensification approach. However, caution must be employed, as many plants in these systems are introduced species.

## Introduction

Agri-environment schemes that support ecological intensification of arable food production often promote interventions such as set-aside, buffer strips and flower-rich field margins as ways to promote pollinator populations and support conservation biocontrol^1^. Many studies, in Europe and North America in particular, have evaluated the impact of such schemes, finding usually—but not always—positive benefits^1,2^. For smallholder farmers ecosystem services are a particularly major input to support crop production, especially where they are unable to afford synthetic interventions such as pesticides, herbicides and fertilisers. Consequently, if field margins and other areas of semi-natural habitat, whether larger (forest remnants) or smaller (field margins), serve as net donors of insect ecosystem services in these regions, the potential benefits to farmers, in addition to the environmental and biodiversity benefits, are considerable.

Non-crop field margin plants and hedgerows provide food, shelter, and breeding sites/larval host plants for a variety of insects beneficial to food production^3–5^. Studies in temperate countries suggest that beneficial insects move from field margins into the crop, providing pollination and natural pest regulation with varying evidence from both European and Asian arable systems^5–8^. However, benefits are not guaranteed as they depend on habitat structure and the associated insect and plant taxa^9^.

In sub-Saharan Africa, however, there is less evidence that beneficial insects move from the margin into the crop^10^. While positive impacts of non-crop vegetation have been observed for pollinators^11^ and natural enemies^12^, other studies found no such impacts^13^. In South African vineyards, for example, while older fields supported high plant and prey diversity and consequently natural enemies, adjacent fynbos remnants did not promote parasitoid abundance within the crop^14^. Smallholder pigeonpea farms in areas of Malawi with more agricultural land relative to non-crop land-use had higher bee abundance^15^. Several hypotheses have been proposed for why field margins do not always confer benefits in terms of natural pest regulation^16^ including insufficient non-crop habitat provision, inadequate populations of suitable natural enemies or detrimental agricultural practices preventing biocontrol establishment; these may equally apply to pollinators in some systems.

The structure, quality and complexity of field margins is highly variable and this, too, can influence the dynamics of beneficial insects using them^17^. It is often assumed that increased botanical diversity, whether as herbs/forbs, shrubs or trees, will automatically increase insect functional and species diversity and consequent ecosystem services such as pollination and pest regulation. This is supported in some European studies^18^ and a limited number of African studies^11^. However, the link between plant diversity and insect ecosystem services is, like the benefit of field margins, not always realised in field systems. For example, in Ethiopian home-gardens, lower rates of predation by arthropods were observed in habitats with high tree diversity compared to low tree diversity^19^.

Many field margins in temperate systems are sown with wildflower mixtures^7^, and sometimes these can include non-native plant species. Similarly in some African agricultural regions, introduced plants often feature in the margins of fields^13^. While exotic species can often provide abundant nectar and pollen for flower-visiting insects^20^, the benefits to individual beneficial insect species can be variable, often favouring generalists over specialists^21^ and common species over rarer ones. This can be due to nutritional insufficiencies of introduced plants’ nectar and pollen^22^, or reduced attractiveness to non-coevolved fauna. Consequently, it is not clear whether margins with a high percentage of exotic species provide the same benefits as those comprising native species only; some studies indicate non-native plants are less beneficial^13^.

A neglected feature of tropical agroecosystems is the diversity of trees present, especially non-forest trees and those that stand singly as boundary markers and landmarks. Recent work in West Africa has shown tree richness supports pollination services in some agroforestry systems in that region^23^ but in arable food crops in East Africa the influence on beneficial insects is not well understood. With up to 54% of farms in Tanzania and around 23% in Malawi having at least one tree, these are a source of fruit and nut crops, timber, fuel, and other valued products^24^. Smallholder farmers usually have a positive attitude towards tree planting^25^. Flowering trees in the tropics provide important forage for insects, as they have long flowering periods, in some cases during the dry season when few other plants flower. They may also provide nesting or sheltering sites for some pollinating species, e.g. dead wood, holes in the trunk, spaces underneath the bark, or around the roots. Further ecosystem benefits may include shade, shelter, soil structure/reduced leaching, habitat for epiphytes, forage etc. which benefit pollinators indirectly. Leguminous trees in particular can support soil quality improvement via fixing nitrogen. Consequently, they have high potential to support beneficial insects and sustainable yields.

We evaluated the relationship between botanical diversity in and around common bean (*Phaseolus vulgaris* L.) fields and invertebrate abundance and activity in smallholder agroecosystems. Common beans are a key protein source for smallholder farmers in this region, and insect pollination provides measurable yield benefits^10,26^, so optimising beneficial insects has considerable potential for livelihood improvement. The crop is mainly considered to be pollinated in African agricultural landscapes by honeybees (*Apis mellifera*) and wild carpenter bees (*Xylocopa* spp.)^27^. Specifically, we sought to test the following hypotheses:

1. Field margins that are botanically rich, rich in native species and rich in tree species are associated with fields that exhibit:
  - Higher abundance and functional diversity of flower-visiting insect taxa
  - Higher abundance of natural enemy taxa
  - Higher visitation rates by flower visiting taxa on the bean crop
2. High species richness of flowering plants when crops are not flowering support higher abundance of natural enemies and functional diversity of flower-visitors in crops.
3. Tree species-rich sites support more robust and connected flower-visitor networks (ultimately fostering greater resilience in pollination systems).
4. Particular tree and herb species are associated more often with the presence of flower-visiting insects and natural enemies of pests.
5. Farms with high plant species richness and particularly high tree species richness ultimately benefit by seeing better yields per unit area.

## Results

### Analysis of botanical richness and species co-occurrence relationships with beneficial insects caught in pan traps

We carried out pan-trapping for invertebrates from the field margins and crop on 24 common bean farms in Tanzania, across three elevation zones on the slope of Mt Kilimanjaro, and 8 farms in Malawi, at the different plant development stages throughout the cropping cycle. A total of 3433 invertebrates categorised as “beneficial” were caught from the pan traps in Tanzania (out of a total of 13,961), and 879 from Malawi (out of 4563). In total, 62 plant species or morphospecies were recorded from Tanzania and 50 from Malawi using quadrats placed along the same transects as the pan traps. The four sites in Malawi identified as having low plant diversity margins nonetheless contained a mean of 8 species of plant per site, compared to a mean of 11 species per site for those with plant species-rich margins (defined as having some non-cultivated habitat along at least one margin). For Tanzania, the mean was higher, 13 plant species per site (with the low-, mid- and high-elevation zones typically having 14, 15 and 10 species per site, respectively).

Table 1a lists the plant species that were most predictive of high trap catches of honeybees, carpenter bees, beeflies, and miscellaneous small bees, respectively. Several tree species, especially *Grevillea robusta* and *Albizia schimperiana*, were associated with high catches of potential pollinators in Tanzania, as was the crop cassava (*Manihot esculenta*) and the herbaceous plants *Acanthospermum hispidum*, and *Euphorbia* sp. Of the plant species associated with presence of the four potential pollinator guilds in Tanzania, 75% were introduced species and 33% of the most strongly associated plants were trees. In Malawi, by contrast, most plant species that were associated with higher trap catches of honeybees, beeflies and small bees were native herbaceous weeds (Table 1b).

**Table 1:** The top 5% of non-ubiquitous, non-scarce plant species most regularly associated with each major potential pollinator guild for common bean in (a) Tanzania and (b) Malawi.

Honeybee	Carpenter bee	Beefly	Other small bees
**(a)**			
*Acalypha fruticosa*	*Drymaria cordata*^2^^?^	*Acanthospermum hispidum*^2^	*Acanthospermum hispidum*^2^
*Acanthospermum hispidum*^2^	*Persea americana*^1,2^	*Euphorbia* sp*.*^2^	*Euphorbia* sp*.*^2^
*Duranta* sp. ^2^	*Tridax procumbens*^2^	*Grevillea robusta*^1, 2^	*Grevillea robusta*^1, 2^
*Pilea tetraphylla*	*Duranta* sp. ^2^	*Manihot esculenta*^2^	*Albizia schimperiana*^1^
*Digitaria* sp.	*Rumex abyssinicus*	*Vachellia (Acacia) tortilis*^1^	*Manihot esculenta*^2^
*Solanum tuberosum*^2^	*Pilea tetraphylla*		

Honeybee	Beefly	Other small bees	
**(b)**			
*Acalypha villicaulis*	Unknown species 1C	*Ageratum conyzoides*^2^	
*Parinari curatellifolia*^1^	*Commelina diffusa*	*Aspilia mossambicensis*	
	*Leucas martinicensis*^2^		

^1^Tree species.

^2^Not native to Eastern or Southern Africa.

Note that carpenter bees were not recorded from pan traps during the survey period in Malawi, and where the top species was not identifiable from vouchers available, the next most commonly associated plant species is also included.

The plant species in Tanzania most strongly associated with high trap catches of the natural enemy guilds (wasps, predatory beetles, hoverflies and spiders) were similarly *G. robusta*, cassava, *A. hispidum* and *Euphorbia* sp*.* (Table 2a). In Malawi, the most commonly associated plant species were *Indigofera spicata*, *Brachystegia stipulata* and *Tithonia diversifolia* for these taxa (Table 2b).

**Table 2:** The top 5% of non-ubiquitous, non-scarce plant species most regularly associated with some major natural enemy guilds for common bean in (a) Tanzania and (b) Malawi.

Wasps	Predatory beetles	Hoverflies	Spiders	Long-legged flies
**(a)**				
*Acanthospermum hispidum*^2^	*Acanthospermum hispidum*^2^	*Acanthospermum hispidum*^2^	*Acanthospermum hispidum*^2^	*Acanthospermum hispidum*^2^
*Euphorbia* sp*.*^2^	*Albizia schimperiana*^1^	*Albizia schimperiana*^1^	*Euphorbia* sp*.*^2^	*Vachellia (Acacia) tortilis*^1^
*Grevillea robusta*^1, 2^	*Euphorbia* sp*.*^2^	*Grevillea robusta*^1,2^	*Grevillea robusta*^1,2^	*Euphorbia* sp*.*^2^
*Manihot esculenta*^2^	*Grevillea robusta*^1, 2^	*Vachellia (Acacia) tortilis*^1^	*Achyranthes aspera*	*Manihot esculenta*^2^
*Vachellia (Acacia) tortilis*^1^	*Leucas* sp.^2^	*Euphorbia* sp*.*^2^	*Manihot esculenta*^2^	*Boerhavia diffusa*
			*Boerhavia diffusa*	
**(b)**				
*Indigofera demissa*	*Brachystegia stipulata*^1^	*Brachystegia stipulata*^1^	*Acalypha villicaulis*	*Indigofera demissa*
*Tithonia diversifolia*^2^	*Triumfetta rhomboidea*^2^	*Trichodesma zeylanicum*^2^	*Bidens pilosa*	*Tithonia diversifolia*^2^

^1^Tree species.

^2^Believed not native to Eastern or Southern Africa.

A canonical correspondence analysis (CCA) for Tanzanian flower-visitors indicated an assemblage of plants including *Senna spectabilis* and *Ocimum gratissimum* were associated with honeybee, carpenter bee and wasp abundance. The picture was more complex in Malawi with different pollinator groups associated with different plant assemblages (Fig. S3 and Supplementary results).

### Evaluation of factors predicting beneficial insect abundance, diversity and activity

Transect walks were performed along one edge of each field while the bean crop was in flower, recording insect visits to flowers on both crop and non-crop plants adjacent to the transect. The most common taxon of potential pollinators recorded from the transect data was the honeybee *A. mellifera*, a medium-sized generalist pollinator, making up 50.2 ± 4.9% (mean ± s.e.m.) of visits to the bean crop flowers. Carpenter bees (*Xylocopa* spp.) contributed 15.9 ± 1.9% of the visits to bean flowers. A total of 464 bees were caught in traps across the two countries, of which 16.0% of a total of 405 from Tanzania were honeybees, and 13.6% of a total of 59 from Malawi were honeybees, with most being various solitary species. The most common guild of potential natural enemy found in the traps was “wasps” (including both aculeate and parasitoid species), comprising 7.8% of the total trap catches.

**Figure 1:**
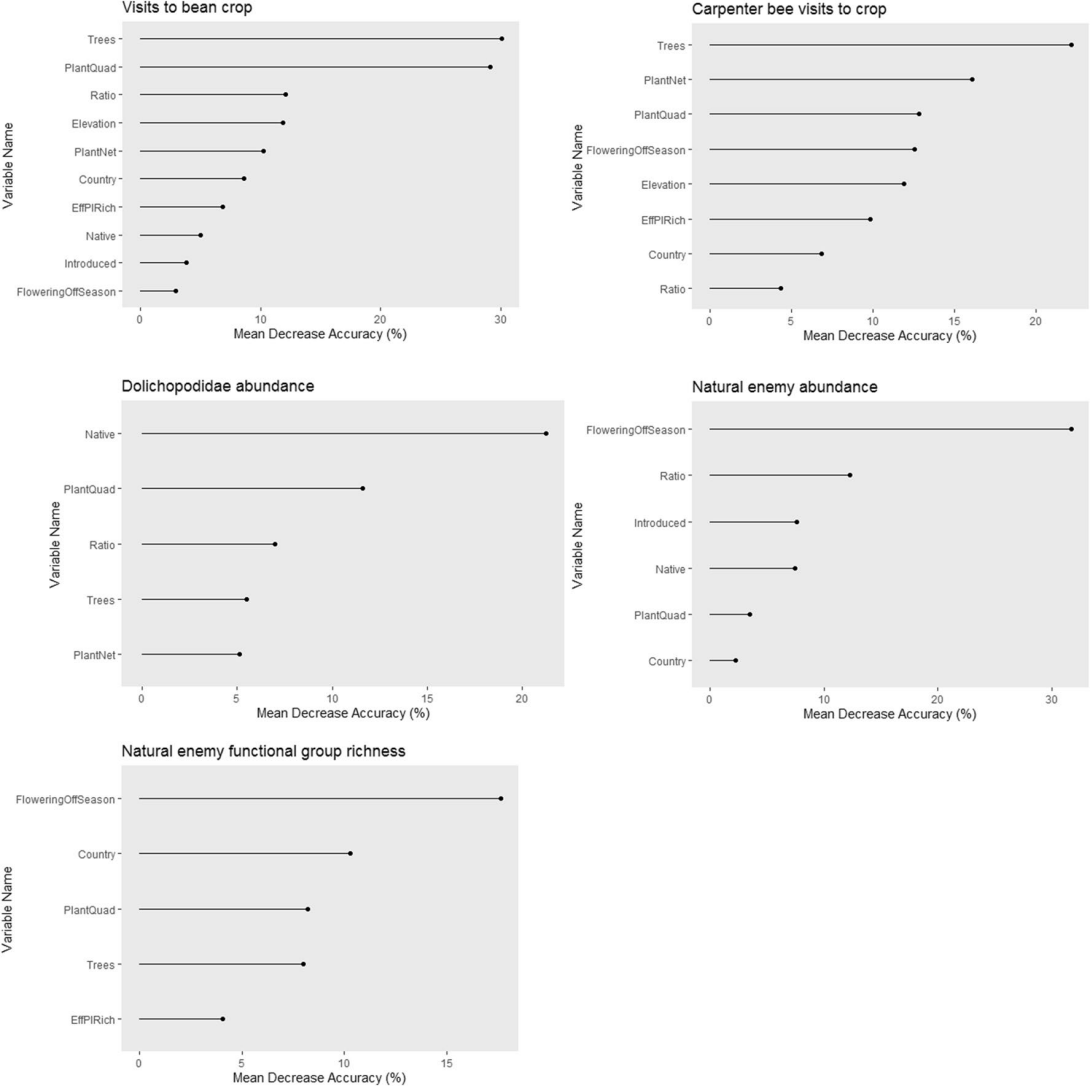
Variable importance plots showing the predictive power of different botanical and site variables on measures of beneficial insect abundance, diversity and activity, as calculated by the random forest models. Mean decrease accuracy indicates the amount by which the model predictive accuracy decreases if a given variable is excluded. Created in R^29^.

**Figure 2:**
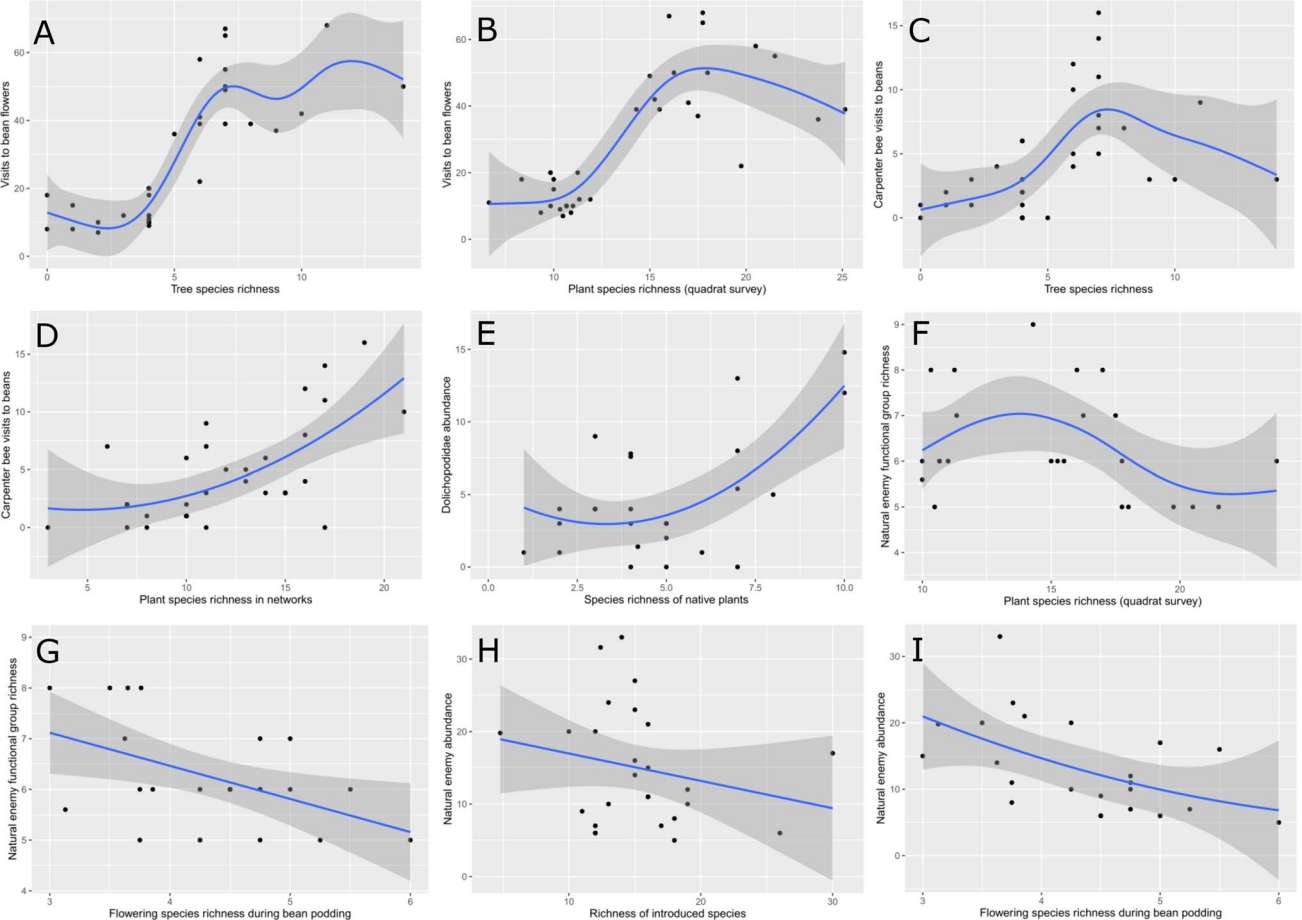
Regressions (where adjusted R^2^ > 0.24) demonstrating the importance of plant and tree richness metrics in predicting pollinator visitation and natural enemy population measures and directionality of relationships. Factors influencing (**A**) and (**B**) all insect visits to beans; (**C**) and (**D**) carpenter bee (*Xylocopa* spp.) visits to beans; (**E**) Dolichopodidae abundance; (**F**) and (**G**) natural enemy functional group richness; and (**H**) and (**I**) natural enemy overall abundance. Created in R^29^ version 3.50 with ggplot2^30^ and edited in GIMP version 2.10.24.

We carried out a Random Forest Model analysis^28^ to identify the most important predictors of high beneficial insect abundance and activity, using a range of explanatory variables related to the location and botanical diversity of fields. This is a machine-learning method for identifying the key predictive variables in a complex data set such as those obtained from ecological surveys, and results are summarised in Table 3. Parameters related to flower visitation were predicted better by the Random Forest Models than were those related to natural enemy trap catches. Variable importance graphs are shown in Fig. 1. Outputs are included in the Supplementary Material (Table S4). For parameters relating to crop visitation by potential pollinators, the most important predictive factors are tree species richness (positively predictive) (Fig. 2A,C), general plant species richness (positively predictive) (Fig. 2B), and richness of plant species involved in interaction networks (positive) (Fig. 2D). Conversely, for natural enemy abundance and richness measures, availability of plants in flower when the bean crop is podding (negative) (Fig. 2G,I) and absolute/relative numbers of native and introduced species (more native species positive; introduced negative) (Fig. 2E,H) are most important, with plant species richness also negatively (but nonlinearly) related to natural enemy functional richness (Fig. 2F).

**Table 3:** Random Forest output summary for main dependent variables.

Output variable	Number of variables used per split in final model	% of variation explained by model	Variables included	Key important variable(s)	%IncMSE (Mean Decrease Accuracy)
Carpenter bee visitation rate to crop (“Carpenter”)	2	49.2	Country, Elevation, PlantNet, PlantQuad, FloweringOffSeason, Ratio, Trees, EffPlRich	Trees	21.17
				PlantNet	18.05
				PlantQuad	12.92
Visits by all insects to crop (“Visits.beans”)	3	73.7	All	PlantQuad	30.21
				Trees	29.88
Dolichopodidae abundance in traps (“Dolichopodidae”)	3	17.01	PlantNet, PlantQuad, Native, Ratio, Trees	Native	21.83
Functional group richness of natural enemies in traps (“FGRich”)	2	12.67	Country, PlantQuad, FloweringOffSeason, Trees, EffPlRich	FloweringOffSeason	17.40
Natural enemy abundance in traps (“NEabundance”)	3	34.1	Country, PlantQuad, FloweringOffSeason, Native, Introduced, Ratio	FloweringOffSeason	31.70
				Ratio	10.41
				Native	9.32

Output variables are defined in the table; for input variables, Country = country location of farm; Elevation = elevation of farm in m asl; PlantNet = number of plant species within network recorded as receiving visits; PlantQuad = species richness at quadrat level (sitewide mean); FloweringOffSeason = species richness of plants in bloom during the podding phase of the crop; Native = native plant species richness; Introduced = introduced plant species richness; Ratio = ratio of native to introduced plants; Trees = mean species richness of trees recorded from quadrat data; EffPlRich = effective plant species number derived from quadrat-level Shannon–Weaver indices.

### Evaluation of network structure

The data from the standardised transect walks enabled construction of plant-flower visitor networks (shown in Supplementary Fig. S4). Those from Tanzania had higher network robustness values for the flower visitor community than networks from Malawi (Fig. S5) (ANOVA, Table S5), indicating that Tanzanian flower visitor networks are likely to be more stable. Conversely, the networks did not differ between countries in connectance, nestedness, interaction evenness or the robustness of the plant community. The tree species richness and the effective plant species richness did not significantly affect the network metrics within either country (Table S6). Across both countries, the most likely effective pollinators of the bean crop (*A. mellifera* and *Xylocopa* spp.) were also recorded on a range of non-crop plants, most frequently for honeybees, *Ageratum conyzoides* and *T. diversifolia* in Tanzania and *Leucas martinicensis* and *Oxygonum sinuatum* in Malawi. Carpenter bees most frequently visited *Desmodium uncinatum* and *D. intortum* in addition to the crop in Tanzania and *O. sinuatum* in Malawi.

### Evaluation of impacts on yield

**Figure 3:**
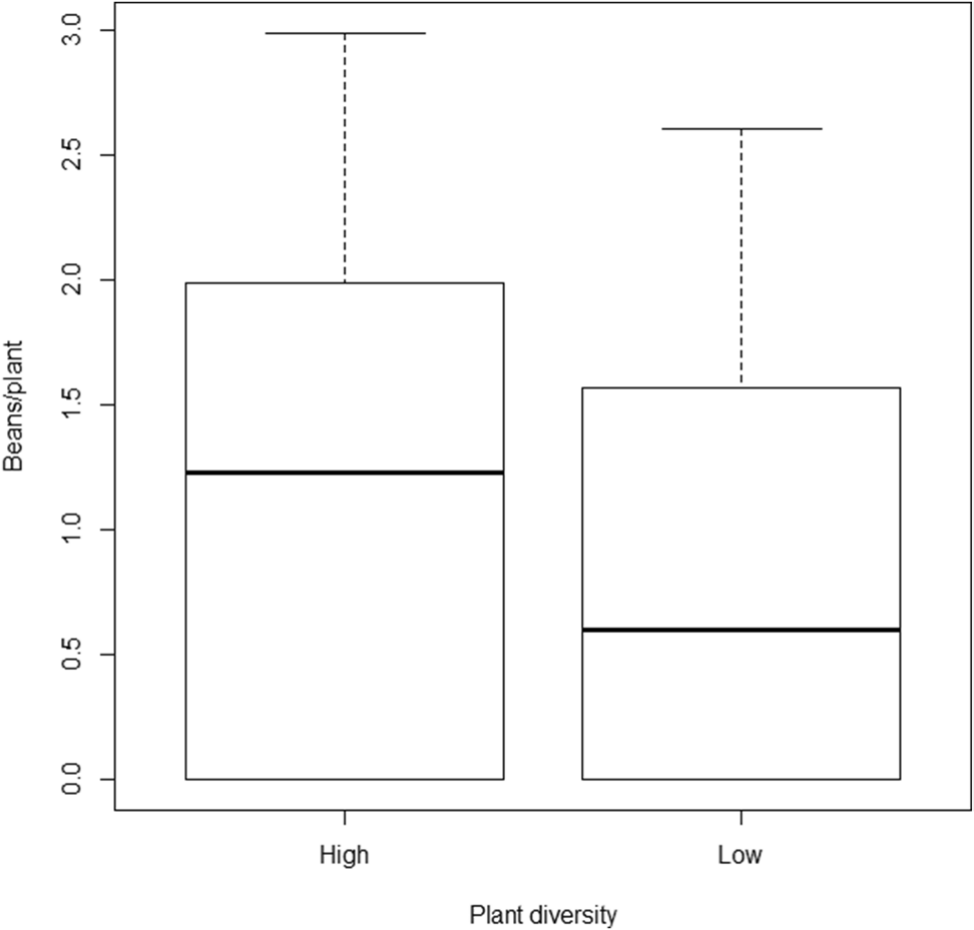
Yield (beans/plant) for the sites in Malawi, categorised according to plant diversity (low = no margin and mean of one or fewer tree species recorded on the transect; high = at least one non-crop vegetated edge to field, mean of 2 or more tree species on the transect). Error bars indicate 1.5*IQR. Created in R^29^ version 3.5.0.

During yield evaluations, the number of beans per plant differed significantly according to the tree species richness on the site (ANOVA, *F*_1_ = 4.80, *p* = 0.031) but not according to the position in the field (ANOVA, *F*_1_ = 0.21, *p* = 0.651), indicating that higher tree species richness is associated with higher yields on farms (Fig. 3).

## Discussion

Trees are often-overlooked within agri-ecosystems. When trees form a component of a mixed system along with diverse herbs and smaller flowering plants, this has been associated with more diverse bee populations in other studies in East Africa^31^ and supported pollination in West African shea agroforestry^23^.

In our study, the presence of trees was strongly associated with higher trap abundance of many key beneficial insects. High tree species richness on a site was also associated with more observed visits by potential pollinators to the crop. Conversely, we found no strong association between tree richness and natural enemies, in contrast to Rezende et al*.*^32^ who linked tree extrafloral nectar to increased natural enemies on farms. However, in Malawi, where yield data were available, tree species richness positively predicted higher crop yields. The role of trees in supporting pollinators and natural enemies in smallholder farming systems is therefore in need of more detailed assessment.

High plant species richness was associated with more visits to crop flowers, including by a key pollinator, as we anticipated. However, contrary to expectations^33^, we found no association between botanical richness on fields and natural enemy abundance or functional group richness. The Kilimanjaro landscape is heterogeneous and well-connected^34^, which may enable more effective movement of natural enemies around and between sites. It is also important to note that this study intended to explore associations in existing smallholdings, rather than infer causality.

The smallholder farms in our study exhibited a high percentage of introduced flora, from regions such as Asia, tropical Americas and Australia, with native plant species sometimes even in a minority. Native plant species richness on the farms was associated with natural enemy abundance, which is likely, in turn, to have a protective effect on the crop^35,36^. Native plants are perhaps more likely to host alternative insect prey/hosts for natural enemies, and so enable populations of natural enemies to thrive^37^. However, native plants are often also more numerous on farms with nearby semi-natural habitat remnants such as woodland fragments, which can contribute to greater natural enemy populations, in which case it may be the habitat fragments rather than the on-farm plants conferring the pest management benefit. Conversely, we found no positive influence of native plant species on the visitation rates by pollinating taxa to the bean crop. This may be because some introduced species provide more abundant nectar over a long flowering period, supporting flower-visitors outside the main season^38^.

The flower visitor networks we constructed indicated that the major pollinators of beans (honeybees and carpenter bees) also visited various other plant species, including both native species (e.g. *O. sinuatum*) and introduced ones (e.g. *D. uncinatum*, *T. diversifolia*, *A. conyzoides*). Several of the highly-visited introduced species have uses as fodder crops, medicinal plants, or as a source of plant-derived pesticides^39–41^; therefore, while they are not part of the native plant community, their presence is likely to be more acceptable to smallholders. However, promotion of any non-native plant cultivation on farms must proceed with particular ecological and social sensitivity.

A high richness of plants in flower during the period that the crop was developing bean pods (“podding” phase) was, contrary to expectations, negatively associated with abundance and diversity of natural enemies. We had predicted that continuous provision of forage (nectar and pollen) would support more stable natural enemy communities year-round and confer pest management benefits. This surprising result indicates there may be ecological interactions (perhaps involving hyperparasitoids) beyond the study’s scope of measurement.

We predicted that plant species-rich sites would support complex, robust and well-connected flower visitor networks; this ultimately fosters greater resilience within pollination systems. However, plant/tree-rich and -poor sites were similar in network metrics; the only factor influencing network parameters was the country being studied, likely due to differences in land-use intensity and field structure.

Pollinator-dependent crops are an important source of micronutrients for many communities in low-income environments, so understanding their pollination ecology is important for food security^42^. Legume crops in sub-Saharan Africa derive considerable yield benefit from robust ecosystem services^26^ and understanding the factors influencing the provision of those services will inform extension services’ recommendations to farmers.

## Conclusions

We identified various plant species associated with beneficial biodiversity on smallholder farms. Many of these species confer multiple ecosystem services: some are fruit trees or vegetable crops (e.g. avocado, cassava), several have pesticidal properties (*T. diversifolia*^43^, *A. conyzoides*^40^) or medicinal properties, and several species are leguminous. Retaining these species in field margins can benefit smallholders in multiple ways. While some are abundantly-flowering nectar resources (e.g. *A. conyzoides*, *A. schimperiana*), other species are small plants with inconspicuous flowers; their relevance in supporting biodiversity is less clear. Some provide habitat (shelter, nesting sites); it is also likely that the presence of some of these species is not causative of high insect abundance, but rather indicative of a shared environmental factor that promotes both the presence of that plant and also the insect populations, such as localised higher moisture levels or shelter from the wind. Understanding the roles of different plants, including native and introduced, and trees, shrubs and forbs, in supporting beneficial biodiversity on farms will support future ecological intensification efforts.

The presence of plant-rich field margins on small farms predicts higher abundance and functional diversity of some beneficial invertebrates. Higher botanical richness is particularly associated with more flower-visitors to bean crops, and native plant richness supports natural enemy abundance. There is a strong case for promoting plant-rich field margins around bean crops on smallholder farms. Several candidate species can be recommended from this study on the basis of positive associations with beneficial invertebrates and their value as pesticidal plants, food or fodder crops in their own right.

Trees in smallholder agro-ecosystems are found to be particularly important, being overrepresented in associations with the presence of key beneficial invertebrates, and consistently and strongly predictive of flower visitor activity. Trees should not be underestimated as components of smallholder farms; the resources they provide for beneficial biodiversity should be thoroughly investigated and promotion and protection of trees on smallholder farms is recommended.

## Materials and methods

### Site selection

Sampling and surveys took place on 24 farm fields in the Moshi Rural district of Northern Tanzania and on 9 farm fields in the Mitundu Extension Planning Area of Malawi (Table S1) (see later details for involvement of specific sites), during the growing seasons of 2015–2017 (Tanzania Year 1 (botanical and trapping surveys): May to September 2016 and September to December 2016 [low/mid], May to October 2016 [high]; Year 2 (transect walks): May to September 2017); Malawi: December 2016 to March 2017). All fields were smallholder farmer fields of < 2 ha sown with local varieties of common beans (*P. vulgaris*), either as the only crop or an intercrop with maize.

Fields were selected from four agro-ecological zones (agriculturally intense mid-elevation in Bunda, Malawi; low elevation zone, Tanzania (< 1000 m); mid elevation zone, Tanzania (1000–1500 m); high elevation zone, Tanzania (> 1500 m)). The three zones in Tanzania represented a gradient of climates and growing conditions^44^ but were all of tropical highland climate with annual rainfall of 600 to 2000 mm (increasing with elevation) and a bimodal rainfall peak^10^. The Malawian zone was of subtropical highland climate with annual rainfall of 700 mm concentrated in a unimodal peak.

Farmers in these regions do use synthetic pesticides and other inputs, at rates that vary between zones but very few use herbicides on the margins^45^. All grew the bean crop according to their normal cultivation practice. They did not mow or burn the margins during the sample period. The farms were selected based on willingness of the farmers to participate, location broadly within a zone, and to be representative of a spectrum of margin types, from minimal (especially in Malawi where cultivation often takes place up to the road edge (Fig. S1)) to highly plant-rich (typical of some Tanzanian fields). Fields were generally walking distance from a village and formed part of a mosaic landscape of fields, settlements and semi-natural areas including wooded graveyards (in Malawi) and uncultivated woodland, wetland and hills^46^.

### Pan trapping—“trap data”

Pan trapping took place as part of a wider study of insect diversity on the farms and the abundance of beneficial insects on each site, particularly because many natural enemies are hard to locate visually without a trapping method^47^. One site per agro-ecological zone was designated as a “primary” site. On these sites, two transects were laid in each field, one running along the field edge, and the other perpendicular to the first, running from the field edge into the centre of the field as described in Mkenda et al*.*^47^. Along the transects were placed five triads of pan traps set 10 m apart. Pan traps were painted white, UV-yellow and blue and were 190 mm in diameter and 110 mm deep (Whitefurze, supplied by Plastic Box Shop, Northallerton, UK). Each pan trap had 300 ml of water added and a drop (around 0.3 ml) of unscented detergent to break the surface tension and improve capture.

Pan traps were deployed once per cropping stage (pre-plough, seedling, flowering, podding, and where possible, post-harvest) in the fields according to the growth cycle of the beans in that zone. This typically ran from March to June and July to September (Tanzania, lower elevations; 2 cycles per year), June to September/October (Tanzania, highest elevations; 1 cycle per year), and November/December to March (Malawi; 1 cycle per year).

Pan traps were left in the field for 48 h, with collections after 24 and 48 h. The content of the pan trap in each case was transferred to a collecting tube (or multiple tubes as needed), transported to the laboratory and preserved in 70% ethanol for later sorting and identification. Insects were identified to functional group level (Table S2), using morphology, with some specimens checked against vouchers in Lilongwe University of Agriculture and Natural Resources collections (Malawi) or Tropical Pesticide Research Institute vouchers (Tanzania) and confirmed at the Royal Botanic Gardens, Kew (UK).

The other sites were designated as “secondary” sites, and a single trap was deployed in the field margin, and a single trap in the crop. Otherwise monitoring proceeded as per the primary sites, and where appropriate means-per-quadrat were used to account for variable sampling effort. Pan trapping was carried out on all 24 sites in Tanzania, and on three of the nine sites in Malawi, for logistical reasons.

### Botanical surveys—“quadrat data”

On all surveyed sites, transects were laid along the margin and perpendicular into the crop, following the same line as the pan traps on primary sites, and analogously to this on secondary sites. Two 1 × 1 m quadrats were placed either side of each pan trap deployed on each site. All plant species present in each quadrat were distinguished at morphospecies level, with vouchers verified against herbarium specimens at the Royal Botanic Gardens, Kew (voucher list available from OSF: https://osf.io/spvda/). The total area (%) of the quadrat occupied by each morphospecies was recorded, and this was repeated at each cropping stage. The flowering status of each species was also recorded, including during the period that the bean plants were developing pods (“podding”) and thus the crop provided no nectar or pollen to insects; this period was used as it could be easily identified for all fields and did not require precise timing around farmers’ planting, harvest or ploughing activities. Plant species were categorised as “native” to the Eastern-Southern African region or “introduced” (typically originating from Asia, tropical Americas or Australia) based on best available information from regional floras, and if the status was unclear (including where species-level identification was uncertain), left unclassified. Botanical surveys were carried out on all sites.

The present study complies with international, national and institutional guidelines. Botanical surveys were conducted with the relevant research permits and permissions in each country. Voucher specimens were handled and transported with the necessary permits according to the local and UK protocols. No CITES species were collected.

### Transect walks—“transect data”

At each cropping stage a standardised transect walk was completed on each field. This followed a similar approach to the BeeWalk methodology^48^ but over a short distance of only 50 m or the length of the field edge, walking at the point where the field and margin meet (Supplementary Fig. S2). All interactions between insects and reproductive parts of any open flower within a 1 m radius of the recorder were noted. Insects were again identified to guild/functional group level (Table S2). If identity was uncertain, insects were captured with a butterfly net and preserved and stored for later identification. The insect identity and the first plant with which it interacted were recorded in each case, enabling construction of interaction networks. Transect walks were carried out on all 24 sites in Tanzania (flower-visitors and natural enemies) and eight of the nine sites in Malawi (flower-visitors only).

### Bean crop yield assessment—“yield data”

In Malawi, yield data was gathered from all eight main farms as part of a pollinator exclusion experiment. This was analysed to test how yield related to plant and tree species richness. Between 8 and 18 bean plants, adjusted for the size of the field and the number of plants within the field were tagged and allowed to be pollinated naturally, and the location (“edge” or “middle” of field) recorded and used in the analysis. When the pods were mature, the number of pods per plant was recorded. Pods were opened and the number of beans per pod counted, to produce a mean beans/pod for each plant. Beans per plant was calculated by multiplying pods per plant by beans per pod.

## Analysis

### Assessment of botanical richness

Effective species richness^49^ was calculated as a measure of the plant diversity for each site derived from the Shannon–Weaver diversity index^50^. These were carried out by calculating the index on a per-quadrat basis for all quadrats on a site, for each survey event, and then calculating the mean site-level diversity value for each site.

### Selection of target beneficial insect groups as indicators

Visit frequency by carpenter bees (*Xylocopa* spp.) was a priority as this genus is a major bean pollinator^27^. Dolichopodidae were selected as a focal natural enemy because the rates of capture of “typical” aphid predators such as lady beetles and lacewings were very low, while parasitoid wasps often degraded in the traps and may be under-recorded. In contrast, Dolichopodidae are known to be predators of aphids and other crop pests^51^ and were abundant, physically robust, and easily identified from traps. The flower visitor data used all Tanzanian sites and all Malawian sites. The natural enemy data used all Tanzanian sites but only two of the Malawian sites due to transport constraints limiting availability of comparable trap data from these sites.

### Analysis of botanical richness and species co-occurrence relationships with beneficial insects caught in traps

To explore the relationship between insects caught in traps and plant species richness/diversity in the immediate surroundings, we calculated contingency coefficients (P_c_) for each trap point and its associated quadrats. These were derived from Eq. (1):

1 $$P_{c} = \, P_{{{\text{plant}}}} \cap P_{{{\text{ins}}}}$$

in which P_plant_ and P_ins_ are the observed frequency of a given plant species and an insect guild respectively, being present at a given transect point. This was compared to the expected co-occurrence (P_pred_) assuming co-occurrence was random. We excluded any plant species that was either found on only one occasion, or was present in 100% of quadrats, as they could not inform our test predictions. We then considered the 5% most commonly associated plant species for each insect guild as most predictive of insect abundance. We carried out a canonical correspondence analysis (CCA) of plant species and insect trap data (full details in the Supplementary Material).

### Evaluation of factors predicting beneficial insect abundance, diversity and activity

In order to identify the most important variables from the sites that explained different parameters related to pollinator activity and natural enemy abundance, Random Forests were run using package ‘randomForest’^28^ (using variables according to Table S3). This methodology has been employed in other cases to extract predictor variables from multivariate data sets, especially with high levels of autocorrelation^52^. Linear, quadratic and general additive models (package ‘mgcv’)^53^ were fitted to data to elucidate the relationships. Full details are provided in the Supplementary Material.

### Evaluation of flower visitor network structure

Networks were constructed from the walked transect data for each site using R version 3.5.0^29^ (via RStudio), employing the package ‘bipartite’^54^. Within countries, sites were classified as above- or below-median tree richness and above- or below-median effective plant species richness, and ANOVAs were used to test whether network connectance, nestedness, interaction evenness and robustness (pollinators) differed between the above- and below-median sites within country.

### Evaluation of impacts on yield

Yield data were analysed by calculating beans/plant and checking for normality. As the data were underdispersed, they were log-transformed before carrying out an analysis of variance with tree species richness and position in field as factors.

## Supplementary Information

Supplementary Information.
