# Supplementary material for: Beneficial insects are associated with botanically rich margins with trees on small farms
Source: Sci Rep. 2021 Jul 26;11:15190. doi: 10.1038/s41598-021-94536-3 (PMC8313545; doi:10.1038/s41598-021-94536-3)
Supplement: Supplementary file 1 — Supplementary Information. [file 41598_2021_94536_MOESM1_ESM.pdf]

Beneficial insects are associated with botanically rich margins with trees on small farms

Sarah E.J. Arnold[1,2]*, Filemon Elisante[2,3], Prisila A. Mkenda[2,4], Yolice L.B. Tembo[5], Patrick A. Ndakidemi[2], Geoff M. Gurr[6], Iain A. Darbyshire[7], Steven R. Belmain[1], Philip C. Stevenson[1,7]

[1]Natural Resources Institute, University of Greenwich, Chatham Maritime, Kent, ME4 4TB, United Kingdom.

[2]The Nelson Mandela African Institution of Science and Technology, Arusha, Tanzania.

[3]Department of Biology, The University of Dodoma, P.O. Box 338, Dodoma, Tanzania.

[4]Biosciences Department, Sokoine University of Agriculture, P.O Box 3000, Morogoro, Tanzania.

[5]Lilongwe University of Agricultural and Natural Resources, Bunda, Lilongwe, Malawi.

[6]School of Agricultural and Wine Sciences, Charles Sturt University, Orange, NSW 2800, Australia.

[7]Royal Botanic Gardens, Kew, Richmond, Surrey, TW9 3AE, United Kingdom.

Supplementary methodology

Canonical Correspondence Analysis

In addition to the co-occurrence coefficients, we carried out a canonical correspondence analysis (CCA) of plant species (from the botanical survey) and insect trap data, using each trap located in the margins as a discrete point. We employed the package 'vegan'[1] in order to explore the predictive relationships between plants and insects on the fields.

Random Forest and General Additive models

For each output variable (listed in Table S3), a Random Forest model was initially built (in R package 'randomForest'[2]) each time using Country + Elevation + PlantNet + PlantQuad + FloweringOffSeason + Native + Introduced + Ratio + Trees + EffPlRich with 2000 trees. The fit was improved by removing variables which showed negative values for % improvement in Mean Squared Error (%IncMSE) values. The number of variables used to split each node (mtry) and the number of trees to build (ntree) was optimised for each model by iteratively plotting the mean squared error for models run with successively different values. The variables were subsequently ranked by importance (Mean Decrease Accuracy: %IncMSE) and the variables that collectively contributed to greater than 50% of the Mean Decrease Accuracy were considered of primary importance. Where no combination of variables met this threshold, the highest %IncMSE variable was considered as the primary predictor. Primary predictor variables were then modelled via a linear, quadratic and general additive fit, with the best model being chosen according to the residual standard error (RSE) and adjusted R^2, with the model producing the lowest RSE taking precedence where the two values suggested different models.

Supplementary results

The CCA for Tanzanian flower-visitors indicated that the assemblage of plants promoting carpenter bee abundance differed from that promoting honeybee abundance (Fig. S2a). In particular, while honeybee abundance was associated with *Vachellia* (syn. *Acacia*) *tortilis*, carpenter bee and small bee abundance was more reliably associated with a plant assemblage including the trees *Senna siamea* and S. *bicapsularis* and the herbs *Ocimum gratissumum* and *Acalypha indica*. In Malawi the CCA revealed that the plant assemblages were not consistently predictive of pollinator abundance *en masse*. The botanical assemblage associated with honeybee and hoverfly abundance was not that which predicted small bee abundance, with honeybees more closely associated with *Acalypha villicaulis* and small bees more closely associated with *Senegalia (Acacia) polyacantha* and *Senna didymobotrya* (Fig. S2b).

Supplementary figures and tables

Fig. S1

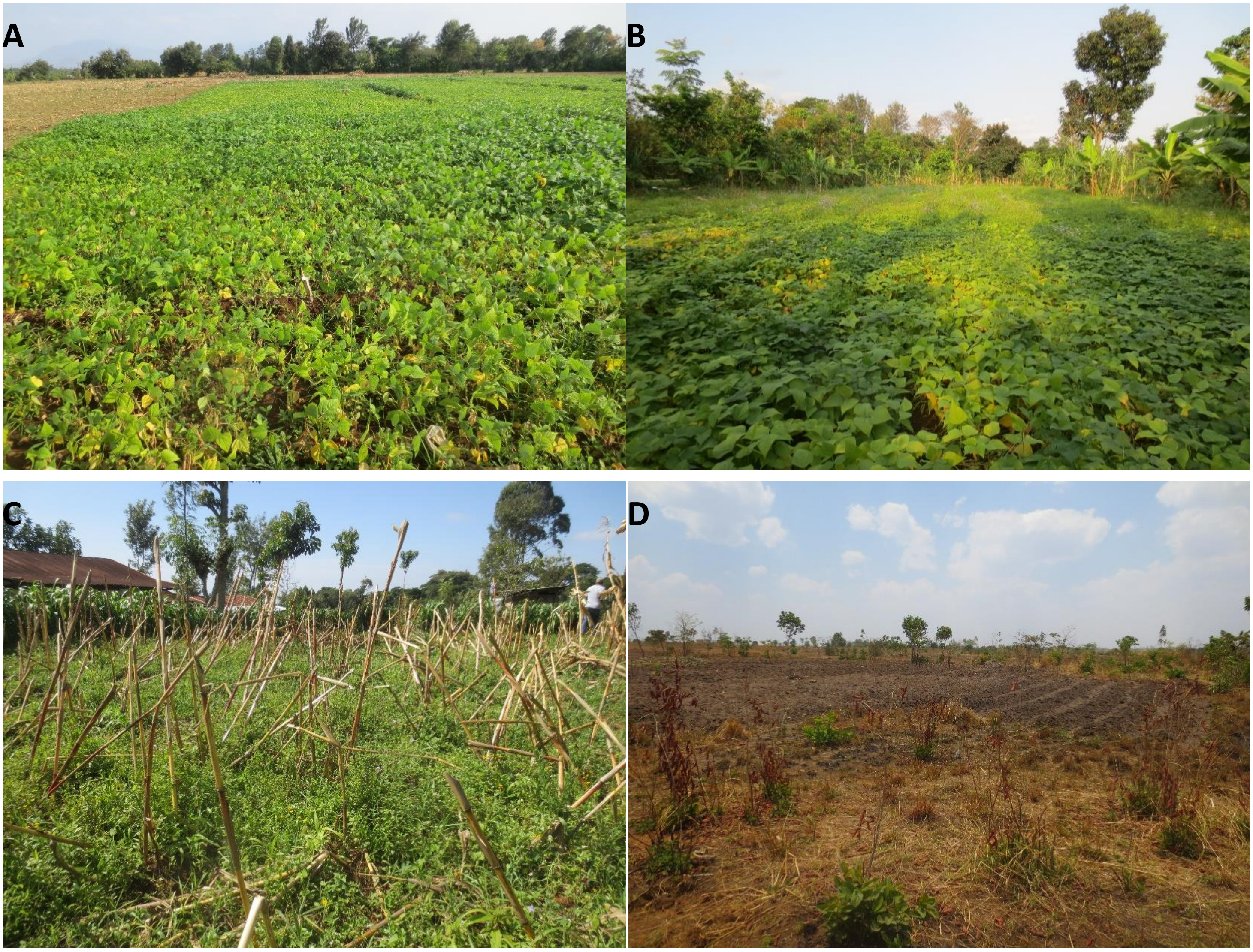

Fig. S1 Examples of typical fields from (A-B) the low and mid zones in Tanzania during the crop podding period, (C) the high zone in Tanzania post-harvest, and (D) Malawi prior to sowing the seed. All photos: SEJ Arnold.

Fig. S2

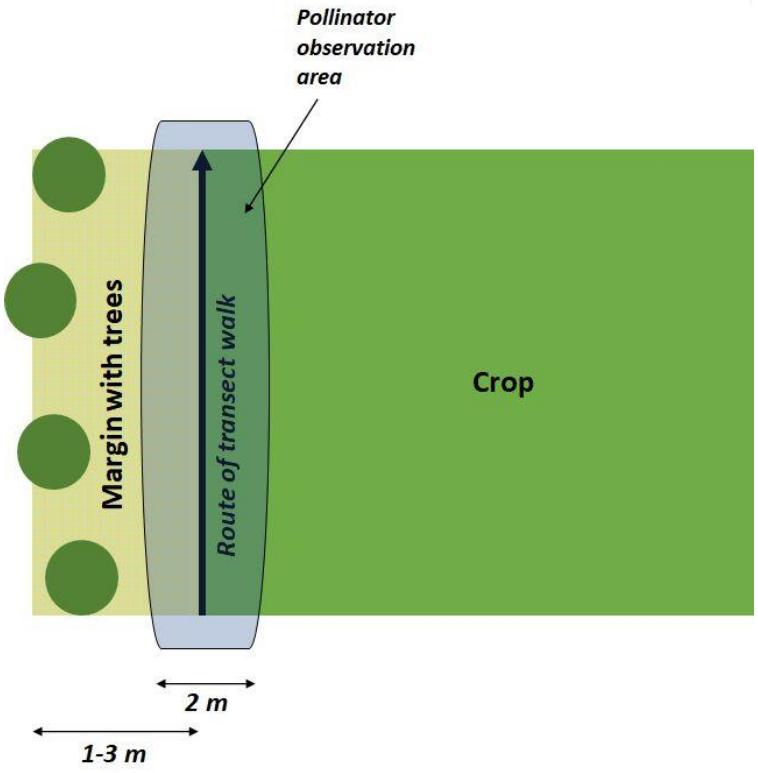

Fig. S2 Pollinator transect walk approach. The walk took place at the interface of the crop (or pre-sowing and post-harvest, the normally cultivated area) and margin, examining an area of 1m to either side and in front of the recorder as they walked along at a slow pace, looking for any interactions between an arthropod and a flower's reproductive organs. Created in MS Powerpoint version 2105.

Figure S3

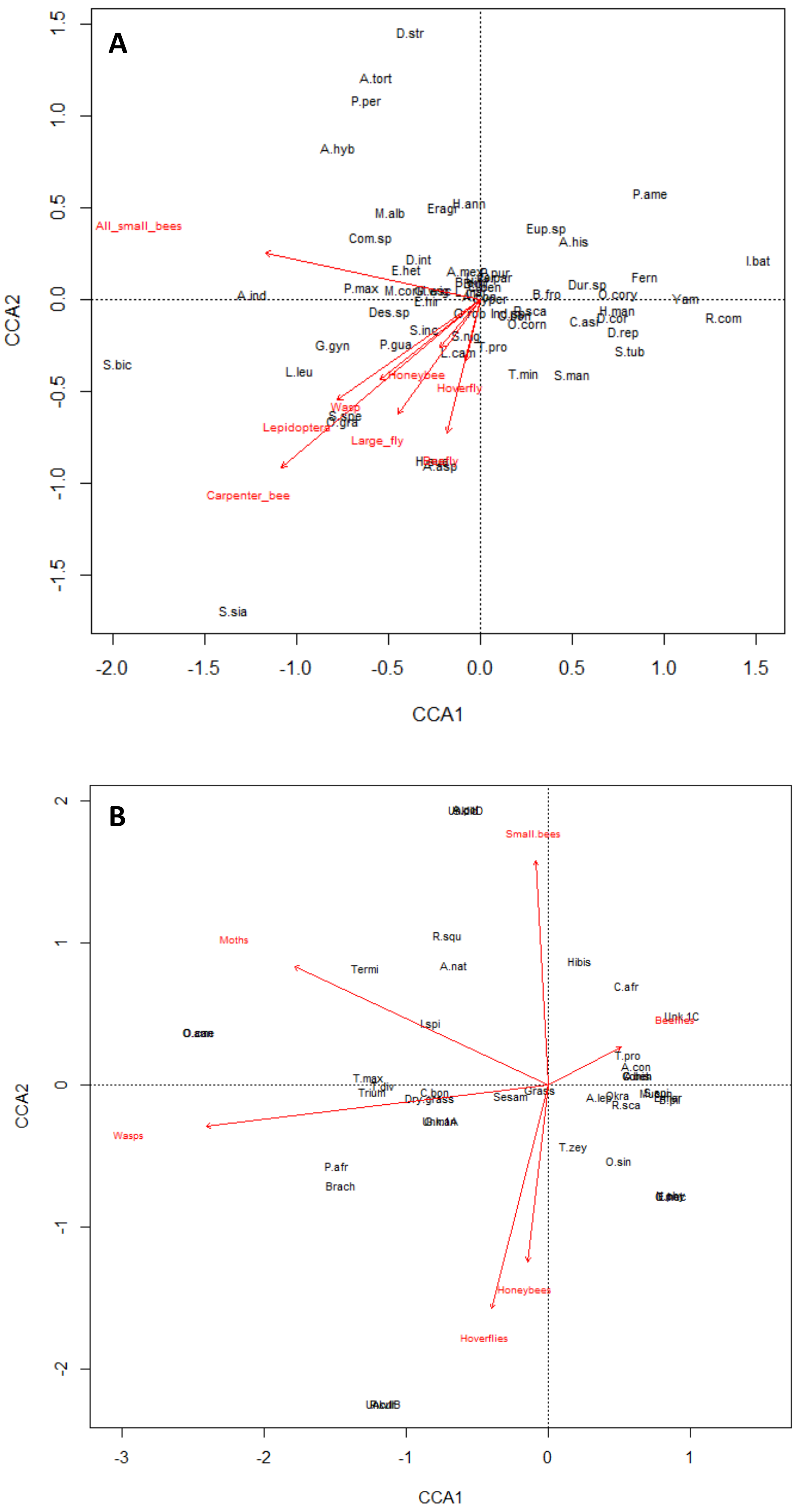
A
D.str
A.tort
P.per
A.hyb
P.ame
M.alb
H.ann
All_small_bees
Eup.sp
A.his
Com.sp
I.bat
D.int
E.het
Fern
Dur.sp
P.max
B.fro
O.cory
A.ind
Yam
H.man
Des.sp
R.com
C.asi
D.cor
O.corn
S.inc
D.rep
G.gyn
P.gua
T.pro
S.tub
S.bic
L.leu
Honeybee
T.min
S.man
Hoverfly
Wasp
Lepidoptera
Large_fly
A.asp
Carpenter_bee
S.sia
CCA1
CCA2
B
Small.bees
R.squ
Moths
Termi
A.nat
Hibis
C.afr
Beetles
I.spi
T.pro
A.con
T.max
Trium
C.bon
Dry.grass
Sesam
Grass
R.sca
Wasps
T.zey
O.sin
P.afr
Brach
Honeybees
Hoverflies
CCA1
CCA2

Fig. S3 Canonical correspondence analysis plots showing relationship between vegetation (black) and guilds of flower-visitors (red) for (a) Tanzania and (b) Malawi. Created in R[3] version 3.5.0 - 3.6.1 using vegan[1].

Abbreviations: Tanzania: A.tort = *Acacia tortilis*; A.ind = *Acalypha indica*; A.his = *Acanthospermum hispidum*; A.asp = *Achyranthes aspera*; A.con = *Ageratum conyzoides*; A.hyb = *Amaranthus hybridus*; A.mex = *Argemone mexicana*; B.fro = *Bidens frondosa*; B.pil = *Bidens pilosa*; B.dif = *Boerhavia diffusa*; C.asi = *Centella asiatica*; C.ben = *Commelina benghalensis*; Com.sp = *Commiphora* sp.; C.bon = *Conyza bonariensis*; Cyper = Cyperaceae; D.str = *Datura stramonium*; D.int = *Desmodium intortum*; Des.sp = *Desmodium* sp.; D.rep = *Dichondra repens*; D.cor = *Drymaria cordata*; Dur.sp = *Duranta* sp.; E.het = *Euphorbia heterophylla*; E.hir = *Euphorbia hirta*; Eup.sp = *Euphorbia* sp.; Fern = Fern; G.par = *Galinsoga parviflora*; G.wig = *Glycine wightii*; G.rob = *Grevillea robusta*; G.gyn = *Gynandropsis gynandria*; H.ann = *Helianthus annuus*; H.man = *Hydrocotyle mannii*; H.sua = *Hyptis suaveolens*; Ind.sp = *Indigofera* sp.; I.bat = *Ipomoea batatas*; L.cam = *Lantana camara*; L.cor = *Launaea cornuta*; L.leu = *Leucaena leucocephala*; L.mar = *Leucas martinicensis*; Eragr = *Eragrostis* sp.; M.esc = *Manihot esculenta*; M.alb = *Morus alba*; M.cor = *Malvastrum coromandelianum*; O.gra = *Ocimum gratissimum*; O.corn = *Oxalis corniculata*; O.cory = *Oxalis corymbosa*; P.max = *Panicum maximum*; P.pur = *Pennisetum purpureum*; P.ame = *Persea americana*; P.per = *Physalis peruviana*; P.gua = *Psidium guajava*; R.sca = *Richardia scabra*; R.com = *Ricinus communis*; S.bic = *Senna bicapsularis*; S.sia = *Senna siamea*; S.spe = *Senna spectabilis*; S.man = *Solanecio mannii*; S.inc = *Solanum incanum*; S.nig = *Solanum nigrum*; S.tub = *Solanum tuberosum*; T.min = *Tagetes minuta*; T.pro = *Tridax procumbens*; Yam = Yam; Malawi (also as for Tanzania): A.pol = *Acacia polyacantha*; A.vil = *Acalypha villicaulis*; A.his = *Acanthospermum hispidum*; P.afr = *Prunus africana*; A.lep = *Thesium schweinfurthii*; A.nat = *Aspilia mossambicensis*; Brach = *Brachystegia stipulata*; C.muc = *Cissampelos mucronata*; C.afr = *Commelina diffusa*; C.ben = *Commelina diffusa*; Corch = *Corchorus* sp.; Dry.grass = Dry grass; G.man = *Gardenia mannii*; Grass = Grass; Hibis = *Hibiscus* sp.; I.spi = *Indigofera demissa*; Mucun = *Neorautanenia mitis*; N.phy = *Nicandra physalodes*; O.ame = *Ocimum americanum*; O.can = *Ocimum canum*; Okra = Okra; O.sin = *Oxygonum sinuatum*; P.cur = *Parinari curatellifolia*; R.squ = *Ruellia squarrosa*; S.spi = *Strychnos spinosa*; S.did = *Senna didymobotrya*; Sesam = *Sesamum* sp.; Termi = *Terminalia* sp.; T.max = *Thysanolaena maxima*; T.div = *Tithonia diversifolia*; T.zey = *Trichodesma zeylanicum*; Trium = *Triumfetta rhomboidea*; Unk-1A = Unknown 1A; Unk-1B = Unknown 1B; Unk-1C = Unknown 1C; Unk-1D = Unknown 1D.

Fig. S4

a)

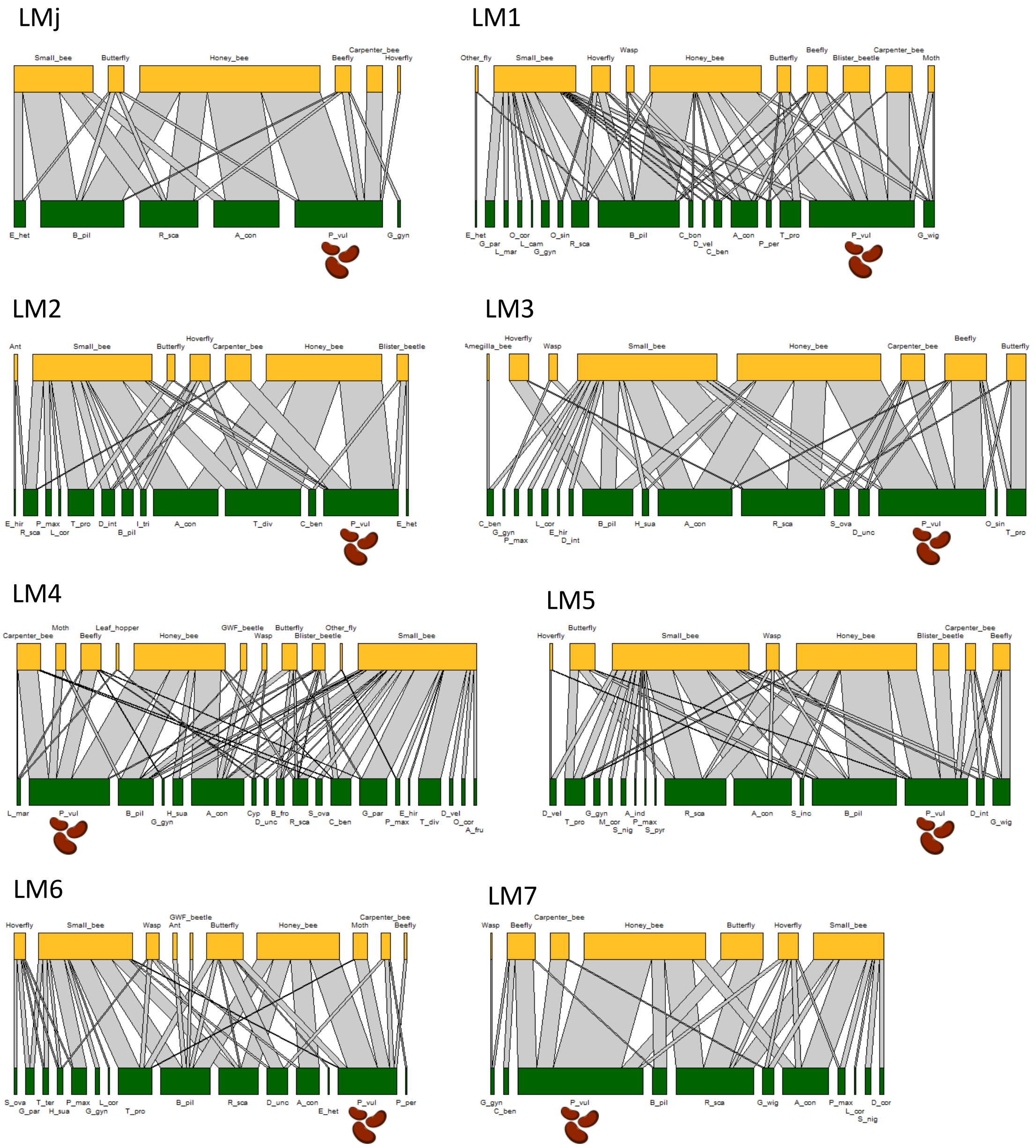
LMj
Small_bee
Butterfly
Honey_bee
Beefly
Carpenter_bee
Hoverfly
E_het
B_pil
R_sca
A_con
P_vul
G_gyn
LM1
Other_fly
Small_bee
Hoverfly
Wasp
Honey_bee
Butterfly
Beefly
Blister_beetle
Carpenter_bee
Moth
E_het
G_par
L_mar
O_cor
L_cam
O_sin
G_gyn
R_sca
B_pil
C_bon
D_vel
C_ben
A_con
P_per
T_pro
P_vul
G_wig
LM2
Ant
Small_bee
Butterfly
Hoverfly
Carpenter_bee
Honey_bee
Blister_beetle
E_hir
R_sca
P_max
L_cor
T_pro
D_int
I_tri
B_pil
A_con
T_div
C_ben
P_vul
E_het
LM3
Amegilla_bee
Hoverfly
Wasp
Small_bee
Honey_bee
Carpenter_bee
Beefly
Butterfly
C_ben
G_gyn
P_max
L_cor
E_hir
D_int
B_pil
H_sua
A_con
R_sca
S_ova
D_unc
P_vul
O_sin
T_pro
LM4
Carpenter_bee
Moth
Beefly
Leaf_hopper
Honey_bee
GWF_beetle
Wasp
Butterfly
Blister_beetle
Other_fly
Small_bee
L_mar
P_vul
B_pil
G_gyn
H_sua
A_con
Cyp
D_unc
B_fro
R_sca
S_ova
C_ben
G_par
P_max
E_hir
T_div
D_vel
O_cor
A_fru
LM5
Hoverfly
Butterfly
Small_bee
Wasp
Honey_bee
Blister_beetle
Carpenter_bee
Beefly
D_vel
T_pro
G_gyn
M_cor
S_nig
A_ind
P_max
S_pyr
R_sca
A_con
S_inc
B_pil
P_vul
D_int
G_wig
LM6
Hoverfly
Small_bee
Wasp
GWF_beetle
Ant
Butterfly
Honey_bee
Moth
Carpenter_bee
Beefly
S_ova
G_par
T_ter
H_sua
P_max
G_gyn
L_cor
T_pro
B_pil
R_sca
D_unc
A_con
E_het
P_vul
P_per
LM7
Wasp
Beefly
Carpenter_bee
Honey_bee
Butterfly
Hoverfly
Small_bee
G_gyn
C_ben
P_vul
B_pil
R_sca
G_wig
A_con
P_max
L_cor
S_nig
D_cor

b)

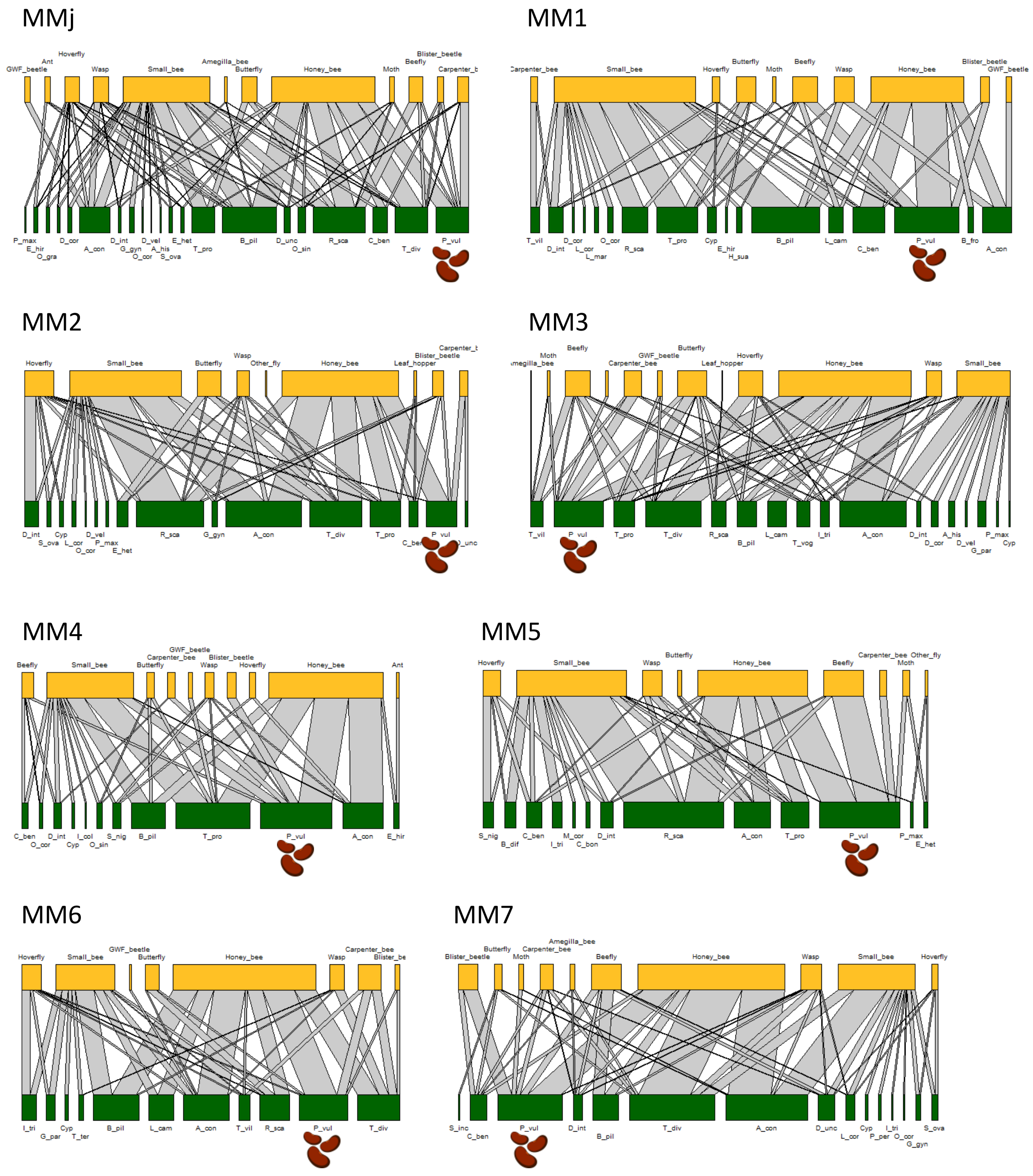

c)

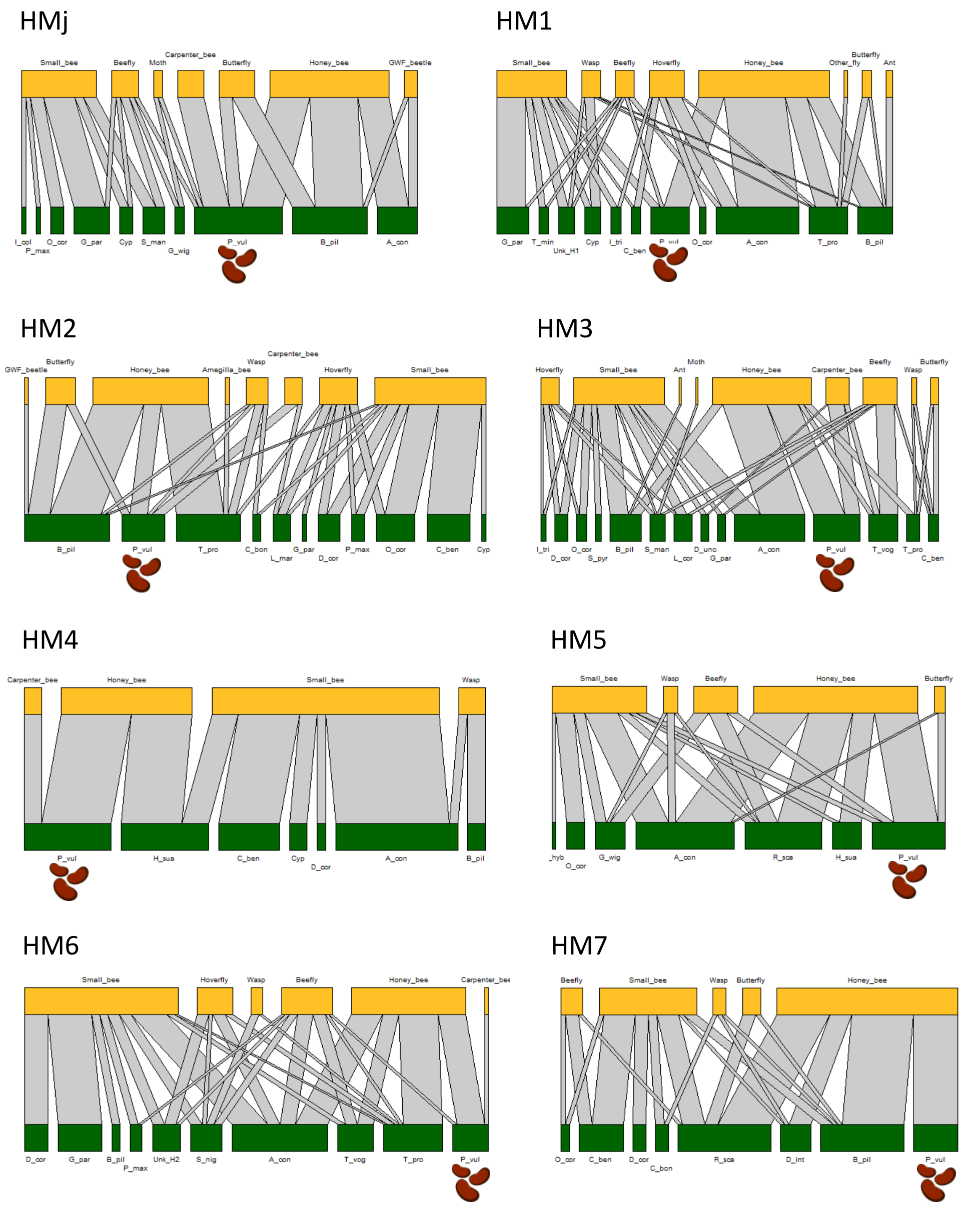
HMj
Small_bee
Beefly
Moth
Carpenter_bee
Butterfly
Honey_bee
GWF_beetle
P_vul
B_pil
A_con
HM1
Small_bee
Wasp
Beefly
Hoverfly
Honey_bee
Butterfly
Other_fly
Ant
G_par
T_min
Unk_H1
Cyp
I_tri
C_ben
O_cor
A_con
T_pro
B_pil
HM2
GWF_beetle
Butterfly
Honey_bee
Amegilla_bee
Wasp
Carpenter_bee
Hoverfly
Small_bee
B_pil
P_vul
T_pro
C_bon
L_mar
G_par
D_cor
P_max
O_cor
C_ben
Cyp
HM3
Hoverfly
Small_bee
Ant
Moth
Honey_bee
Carpenter_bee
Beefly
Wasp
Butterfly
I_tri
D_cor
O_cor
S_pyr
B_pil
S_man
L_cor
D_unc
G_par
A_con
P_vul
T_vog
T_pro
C_ben
HM4
Carpenter_bee
Honey_bee
Small_bee
Wasp
P_vul
H_sua
C_ben
Cyp
D_cor
A_con
B_pil
HM5
Small_bee
Wasp
Beefly
Honey_bee
Butterfly
O_cor
G_wig
A_con
R_sca
H_sua
P_vul
HM6
Small_bee
Hoverfly
Wasp
Beefly
Honey_bee
Carpenter_bee
D_cor
G_par
B_pil
P_max
Unk_H2
S_nig
A_con
T_vog
T_pro
P_vul
HM7
Beefly
Small_bee
Wasp
Butterfly
Honey_bee
O_cor
C_ben
D_cor
C_bon
R_sca
D_int
B_pil
P_vul

d)

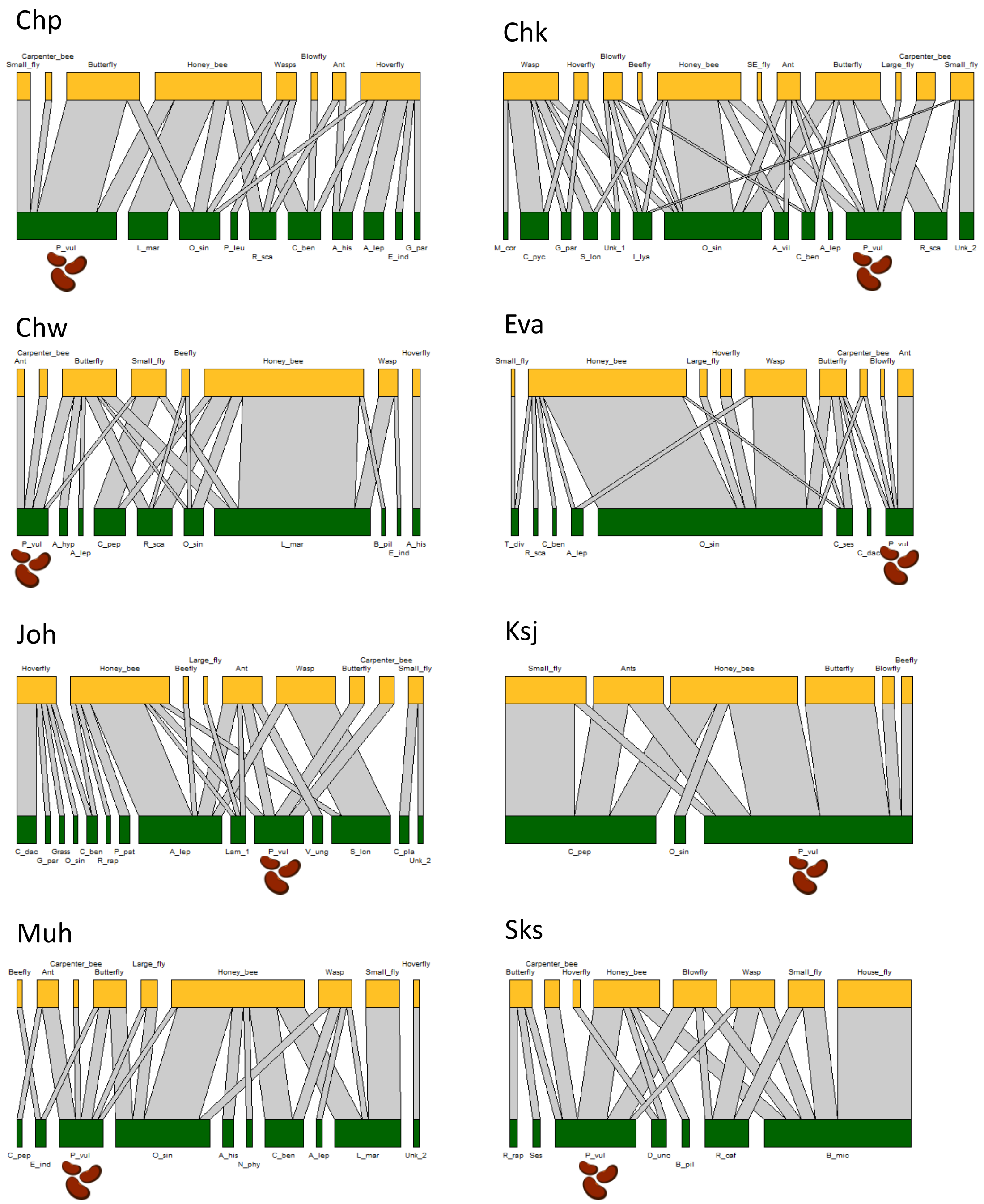

Fig. S4 Flower visitor network diagrams for the full sampling period for the individual sites for (a) the low zone, (b) the mid zone, (c) the high zone in Tanzania, and (d) Malawi. Created in R[3] version 3.5.0 using bipartite[4] and modified in MS Powerpoint version 2105.

Abbreviations: Tanzania: A_con = *Ageratum conyzoides*; A_fru = *Acalypha fruticosa*; A_his = *Acanthospermum hispidum*; A_hyb = *Amaranthus hybridus*; A_ind = *Acalypha indica*; B_dif = *Boerhavia diffusa*; B_fro = *Bidens frondosa*; B_pil = *Bidens pilosa*; C_ben = *Commelina benghalensis*; C_bon = *Conyza bonariensis*; C_pep = *Cucurbita pepo*; Cyp = Cyperaceae; D_cor = *Drymaria cordata*; D_int = *Desmodium intortum*; D_unc = *Desmodium uncinatum*; D_vel = *Digitaria velutina*; E_het = *Euphorbia heterophylla*; E_hir = *Euphorbia hirta*; G_gyn = *Gynandropsis gynandria*; G_par = *Galinsoga parviflora*; G_wig = *Glycine wightii*; H_sua = *Hyptis suaveolens*; I_col = Indigofera colutea; I_tri = *Indigofera trita*; L_cam = *Lantana camara*; L_cor = *Launaea cornuta*; L_mar = *Leucas martinicensis*; M_con = *Malvastrum coromandelianum*; O_cor = *Oxalis corniculata*; O_gra = *Ocimum gratissimum*; O_sin = *Oxygonum sinuatum*; P_max =

Panicum maximum; P_per = *Physalis peruviana*; P_ vul = *Phaseolus vulgaris* (crop); R_sca = *Richardia scabra*; S_inc = *Solanum incanum*; S_man = *Solanecio mannii*; S_nig = *Solanum nigrum*; S_ova = *Sida ovata*; S_pyr = *Sporobolus pyramidalis*; T_div = *Tithonia diversfolia*; T_min = *Tagetes minuta*; T_pro = *Tridax procumbens*; T_ter = *Tribulus terrestris*; T_vil = *Tephrosia villosa*; T_vog = *Tephrosia vogelli*; Unk_H1 = Unknown H1; Unk_H2 = Unknown H2; Malawi (also as for Tanzania): A_hyp = *Arachis hypogaea*; A_lep = *Thesium schweinfurthii*; A_vil = *Acalypha villicaulis*; B_mic = *Bridelia micrantha*; C_afr = *Commelina diffusa*; C_dac = *Cynodon dactylon*; C_ pyc = *Chloris pycnothrix*; C_pla = *Combretum platyphyllum*; C_ses = *Ceratotheca sesamoides*; E_ind = *Eleusine indica*; Grass = Grass (not firmly identified); I_lya = *Tephrosia rhodesica*; Lam_1 = Lamiaceae sp.; N_phy = *Nicandra physalodes*; P_leu = *Phyllanthus leucanthus*; P_pat = *Pavonia patens*; R_caf = *Rauvolfia caffra*; R_rap = *Raphanus raphanistrum*; S_lon = *Sansevieria longiflora*; Ses = *Sesamum* sp.; Unk_1 = *Acmella radicans*; Unk_2 = Unknown 2; V_ung = *Vigna unguiculata*.

Figure S5

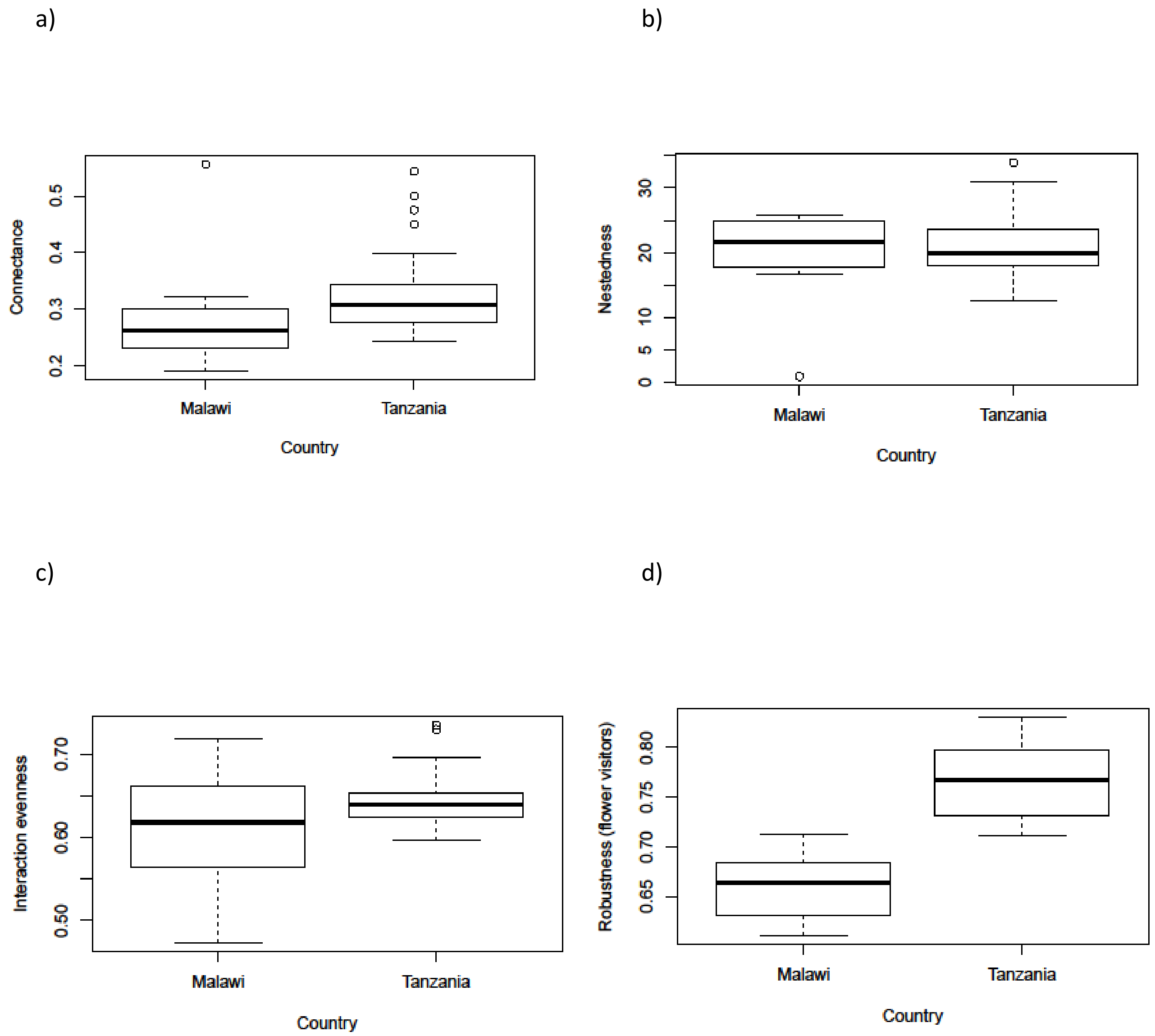

Fig. S5 Network metrics for flower visitor networks from Tanzania and Malawi, showing (a) network connectance; (b) network nestedness; (c) interaction evenness and (d) robustness of the flower visitor community. Connectance, nestedness and robustness differ significantly between the countries, with connectance and robustness being higher in Tanzania and nestedness higher in Malawi. Created in R[3] version 3.5.0.

Table S1. Site list where data were collected.

Site code	Country	Zone	Latitude (°S)	Longitude (°E)	Elevation	Notes
LMj	Tanzania	Low	3.4015	37.5563	827	
LM1	Tanzania	Low	3.4012	37.5563	824	
LM2	Tanzania	Low	3.3481	37.5411	831	
LM3	Tanzania	Low	3.3987	37.5615	833	
LM4	Tanzania	Low	3.3981	37.5614	835	
LM5	Tanzania	Low	3.3982	37.5609	833	
LM6	Tanzania	Low	3.3989	37.5586	831	
LM7	Tanzania	Low	3.3983	37.5584	833	
MMj	Tanzania	Mid	3.3466	37.5435	1002	
MM1	Tanzania	Mid	3.2644	37.5277	1009	
MM2	Tanzania	Mid	3.3453	37.5419	1012	
MM3	Tanzania	Mid	3.3445	37.5425	1017	
MM4	Tanzania	Mid	3.3466	37.5426	1005	
MM5	Tanzania	Mid	3.3446	37.5432	1013	
MM6	Tanzania	Mid	3.3478	37.5412	1002	
MM7	Tanzania	Mid	3.3441	37.5437	1016	
HMj	Tanzania	High	3.2477	37.5025	1853	
HM1	Tanzania	High	3.2629	37.5092	1665	
HM2	Tanzania	High	3.2593	37.5076	1705	
HM3	Tanzania	High	3.2583	37.5076	1712	
HM4	Tanzania	High	3.2518	37.5051	1788	
HM5	Tanzania	High	3.2466	37.5058	1834	
HM6	Tanzania	High	3.2468	37.5044	1846	
HM7	Tanzania	High	3.2463	37.5041	1856	
Chp	Malawi		14.2151	33.7578	1168	
Chk	Malawi		14.2146	33.7546	1163	
Chw	Malawi		14.2122	33.7600	1172	
Eva	Malawi		14.2162	33.7566	1164	
Ksj	Malawi		14.2110	33.7556	1167	
Joh	Malawi		14.2143	33.7528	1158	
Sks	Malawi		14.2124	33.7600	1171	
Muh	Malawi		14.2109	33.7562	1171	
Lng	Malawi		14.2121	33.7551	1148	Limited data
Kmp	Malawi		14.2104	33.7551	1169	Limited data
Kgw	Malawi		14.1936	33.7727	1132	Limited data

Table S2. Functional groups used in the field surveys

Functional group	Taxonomic groups included	Assumed functional significance
Pollinator: Social short proboscis nectar- and pollen-collectors (Honeybees)	*Apis mellifera*	Common visitor to bean flowers, able to manipulate flowers, but pollination efficacy uncertain
Pollinator: Small nectar- and pollen-collectors (Small bees)	*Amegilla* sp., *Ceratina* sp., other Hymenoptera: Apidae	Possible pollinator of beans
Pollinator: Large, long proboscis nectar- and pollen-collectors (Carpenter bees)	*Xylocopa* spp.	Likely important pollinator of beans
Pollinator: Short proboscis pollen-feeders (Hoverflies)	Diptera: Syrphidae	Possible pollinator of beans, natural enemy of bean pests (predation of groups of pests by larvae)
Pollinator: Long-proboscis nectar-feeders with non-pest larvae (Beeflies)	Diptera: Bombylidae	Possible pollinator of beans
Possible Pollinator: Other highly active flying flower visitors	Diptera not otherwise classified	Various
Pollinator/Pest: Long proboscis nectar feeders with pest larvae (Butterflies and moths)	Lepidoptera	Possible pollinator of beans<br>Larvae may be pests
Natural Enemy: Medium and large	Hymenoptera: Vespidae, Hymenoptera: Polistidae, Hymenoptera: Crabronidae	Possible minor pollinator of beans, natural enemy of bean

flying predators (Predatory wasps)		pests (predation of individual pests by adults)
Natural Enemy: Ground-based predators (Predatory beetles)	Coleoptera: Coccinellidae; Coleptera: Staphylinidae	Natural enemy of bean pests (predation of groups of pests by larvae and adults)
Natural Enemy: Small parasitoids (Parasitic wasps)	Hymenoptera: Braconidae and diverse Chalcidoidea	Natural enemy of bean pests (parasitoid)
Natural Enemy: Larger parasitoids (Parasitic flies)	Diptera: Tachinidae	Natural enemy of bean pests and other insects (parasitoid)
Natural Enemy: Small flying predators (Long-legged flies)	Diptera: Dolichopodidae	Natural enemy of bean pests (predation of individual pests by adults)
Pest: Flower feeders (Blister beetles)	Coleoptera:	Pest of bean flowers, but frequent flower visitor so retained for completeness
Possible Natural Enemy: Social non-flying nectar feeders (Ants)	Hymenoptera: Formicidae	Significance uncertain (may be a predator or may be detrimental to natural enemy activity) but frequent flower visitor
Other beetles	Coleoptera not otherwise classified	Various

Table S3. Variables used in Random Forest models

Variable	Input or Output	Type of measure (for output variables)	Origin data set	Code
All insect visits to beans	Output	Flower visitors	Transect	Visits.beans
Carpenter bee visits to beans	Output	Flower visitors	Transect	Carpenter
Natural enemy functional group richness in traps	Output	Natural enemies	Pan traps	FGrich
Natural enemy abundance	Output	Natural enemies	Pan traps	NEabundance
Dolichopodidae abundance	Output	Natural enemies	Pan traps	Dolichopodidae
Plant effective species number	Input	-	Quadrat	EffSpRich
Plant species richness (per quadrat)	Input	-	Quadrat	PlantQuad
Plant species richness (being visited in networks)	Input	-	Transect	PlantNet
Tree species richness	Input	-	Quadrat	Trees
Species richness of plants blooming during the bean podding period	Input	-	Quadrat	FloweringOffSeason
Native plant species richness	Input	-	Quadrat	Native
Introduced plant species richness	Input	-	Quadrat	Introduced
Ratio of native to introduced plant species	Input	-	Quadrat	Ratio

Table S4. Linear and non-linear results for the relationship between explanatory variables ranked highly by the Random Forest models, and the key output variables. A larger 'Ratio' value is indicative of more introduced species relative to native species.

Output variable	Explanatory variable	Optimal model (lin = linear; quad = quadratic; gam = general additive)	Sample size	Adjusted R^2	General directionality of relationship
Visits.beans	Trees	gam	32	0.8160	Positive
Visits.beans	PlantQuad	gam	32	0.7620	Positive
Visits.beans	Ratio	quad	32	0.2562	Positive
Carpenter	Trees	gam	32	0.5380	Positive
Carpenter	PlantNet	quad	32	0.3762	Positive
Carpenter	PlantQuad	quad	32	0.3095	Positive
Dolichopodidae	Native	quad	26	0.3664	Positive
Dolichopodidae	PlantQuad	quad	26	0.1792	Negative
Dolichopodidae	Ratio	lin	26	0.0873	Negative
FGrich	FloweringOffSeason	lin	26	0.2279	Negative
FGrich	PlantQuad	gam	26	0.2870	Complex
NEabundance	FloweringOffSeason	quad	26	0.4499	Negative
NEabundance	Ratio	poly	26	0.0772	Negative
NEabundance	Introduced	gam	26	0.2490	Negative

Table S5. ANOVA results for the difference in network statistics between Tanzania and Malawi. Asterisks indicate significance at * = 0.05, ** = 0.01, *** = 0.001 level.

Dependent variable	Independent variable	F-value	df	p-value
Connectance	Country	1.112	1	0.300
Interaction evenness	Country	3.543	1	0.070
Nestedness	Country	0.565	1	0.458
Robustness (flower visitors)	Country	55.135	1	<0.001 ***
Robustness (plants)	Country	3.884	1	0.058

Table S6. Results of ANOVAs examining network metrics (using linear models testing Variable~Country/RichnessVariable) in which sites were categorised as above- or below-median for their respective country.

Network metric	*F*-value Country/TreeRichness	*p*-value Country/TreeRichness	*F*-value Country/EffSpRich	*p*-value Country/EffSpRich
Connectance	0.8723	0.4290	2.2219	0.1272
Interaction evenness	0.6119	0.54941	0.4147	0.66453
Nestedness	0.9266	0.4077	0.7061	0.5021
Robustness (pollinators)	0.0042	0.9958	1.9225	0.1651

Supplementary references

1 Oksanen, J. *et al.* vegan: Community Ecology Package. R package version 2.5–3. (2018).

2 Liaw, A. & Wiener, M. Classification and regression by randomForest. *R news* **2**, 18-22 (2002).

3 R Core Team. R: A language and environment for statistical computing., https://www.R-project.org/. (2020).

4 Dormann, C. F., Gruber, B. & Fruend, J. Introducing the bipartite Package: Analysing Ecological Networks. *R news* **8/2**, 8-11 (2008).
